# A Student Facial Expression Recognition Model Based on Multi-Scale and Deep Fine-Grained Feature Attention Enhancement

**DOI:** 10.3390/s24206748

**Published:** 2024-10-20

**Authors:** Zhaoyu Shou, Yi Huang, Dongxu Li, Cheng Feng, Huibing Zhang, Yuming Lin, Guangxiang Wu

**Affiliations:** 1 School of Information and Communication, Guilin University of Electronic Technology, Guilin 541004, China; guilinshou@guet.edu.cn (Z.S.); 22022303042@mails.guet.edu.cn (Y.H.); fengcheng@mails.guet.edu.cn (C.F.); 2 Guangxi Wireless Broadband Communication and Signal Processing Key Laboratory, Guilin University of Electronic Technology, Guilin 541004, China; 3 School of Computer and Information Security, Guilin University of Electronic Technology, Guilin 541004, China; zhanghuibing@guet.edu.cn (H.Z.); ymlin@guet.edu.cn (Y.L.); 4 34th Research Institute of China Electronics Technology Group Corporation, Guilin 541004, China; wuguangxiang@cetc.com.cn

**Keywords:** facial expression recognition, smart classroom, multi-scale features, deep fine-grained features, key region-oriented attention mechanism

## Abstract

In smart classroom environments, accurately recognizing students’ facial expressions is crucial for teachers to efficiently assess students’ learning states, timely adjust teaching strategies, and enhance teaching quality and effectiveness. In this paper, we propose a student facial expression recognition model based on multi-scale and deep fine-grained feature attention enhancement (SFER-MDFAE) to address the issues of inaccurate facial feature extraction and poor robustness of facial expression recognition in smart classroom scenarios. Firstly, we construct a novel multi-scale dual-pooling feature aggregation module to capture and fuse facial information at different scales, thereby obtaining a comprehensive representation of key facial features; secondly, we design a key region-oriented attention mechanism to focus more on the nuances of facial expressions, further enhancing the representation of multi-scale deep fine-grained feature; finally, the fusion of multi-scale and deep fine-grained attention-enhanced features is used to obtain richer and more accurate facial key information and realize accurate facial expression recognition. The experimental results demonstrate that the proposed SFER-MDFAE outperforms the existing state-of-the-art methods, achieving an accuracy of 76.18% on FER2013, 92.75% on FERPlus, 92.93% on RAF-DB, 67.86% on AffectNet, and 93.74% on the real smart classroom facial expression dataset (SCFED). These results validate the effectiveness of the proposed method.

## 1. Introduction

Facial expression recognition plays an important role in the field of education, which is mainly reflected in two aspects: firstly, it helps teachers to assess students’ emotional states and attention levels in real time [1], further adjusting teaching strategies. Secondly, it supports personalized teaching; specifically, teachers can gain insights into their learning situation and needs through analyzing students’ facial expressions and emotional states to provide targeted teaching and counseling [2]. However, in the classroom environment, factors such as lighting variations, viewpoint diversity, and distance differences can severely affect the accuracy of facial expression recognition. Therefore, enhancing the robustness and accuracy of facial expression recognition models to cope with complex classroom environments has become a critical problem to be solved.

In the past few decades, advancements in VR technology have enabled researchers to create specific virtual environments designed to elicit emotional responses, thereby improving the recognition of facial expressions. Castiblanco et al. [3] conducted a study that utilized virtual reality environments combined with electroencephalography (EEG) and machine learning techniques to explore the effectiveness of EEG in emotional representation. The results showed a significant consistency between self-assessments and classifier outcomes, further supporting the feasibility of using EEG as a tool for emotional representation. Marín-Morales et al. [4] proposed an emotion recognition system that evokes emotional states through immersive virtual environments. Based on EEG and electrocardiogram (ECG) data from 60 participants, the system achieved high accuracy using a support vector machine classifier, validating the potential application of immersive virtual environments in emotion recognition. However, despite the progress made in virtual environment research, exploring emotional responses and facial expression recognition in real-world settings remains a significant challenge for researchers. Saurav et al. [5] used the AdaBoost feature selection algorithm to select key facial features from the original HOG features, thereby improving the accuracy of facial expression recognition. Shi et al. [6] addressed the variability in the number and location of key points in the traditional SIFT algorithm by proposing an improved version. This new method optimizes key point positions through shape decomposition and a key point constraint algorithm, while also extracting regional gradient information for emotion recognition. Niu et al. [7] proposed a facial expression recognition algorithm combining ORB and LBP features. The algorithm extracts effective features through face detection, employs region partitioning to enhance computational speed, and finally uses SVM for classifying the combined features to recognize different facial expressions. Lakshmi et al. [8] proposed a modified HOG and LBP feature descriptor for extracting features from the detected eye, nose, and mouth regions, thus recognizing facial expressions. The above studies use traditional manual features to recognize facial expressions in real environments, which saves computational resources. However, these methods rely on manually designed features, which are time-consuming and laborious, and the models have limited recognition accuracy and weak generalization performance. Consequently, in recent years, researchers have gradually shifted their focus to the field of deep learning. Shou et al. [9] proposed a residual channel-crossing transformer mask network to accurately extract the key features of students’ facial expressions. Xue et al. [10] proposed a facial expression recognition network with smooth prediction from coarse to fine. In the first stage, it identifies facial expressions that are relatively similar and categorizes them into the same class. In the second stage, it captures unique fine-grained facial features and reclassifies the expression categories from the first stage. Nan et al. [11] introduced an attention mechanism to enhance the extraction of facial features in the MobileNetV1 model. By combining center loss with softmax loss, they optimized the model parameters to reduce intra-class distance and increase inter-class distance, thereby improving the accuracy of facial expression classification. Farzaneh et al. [12] proposed a deep attention center loss method that enhances discriminative power by adaptively selecting a subset of important feature elements. By embedding an attention mechanism, this method adaptively achieves intra-class compactness and inter-class separation, effectively extracting relevant facial information. Zhang et al. [13] proposed a novel method of rebalancing attention mapping, which regularizes the model to extract transformation-invariant information from minor classes within the training samples. Additionally, they introduced rebalanced smooth labels to adjust the cross-entropy loss, leveraging the label distribution in imbalanced data to improve the model’s performance in imbalanced facial expression recognition (FER) tasks. Yu et al. [14] addressed the limitations of FER dataset size and class imbalance by generating pseudo-labels through semi-supervised learning, employing uniform sampling, and introducing temporal encoders. These strategies significantly improved the accuracy of facial expression recognition.

Although the current facial expression recognition methods based on deep learning have achieved certain results, there are still some shortcomings: (1) existing models ignore the problem of scale variation, which leads to the possibility of losing some key information when extracting facial features, thus misclassifying similar expressions at different scales as different expressions; (2) the extraction of deep fine-grained features is insufficient, making it difficult to distinguish expressions with subtle differences; (3) previous methods mainly rely on the overall or relatively rough local analysis, which only capture limited features and are easily affected by external factors, thereby reducing the accuracy and robustness of recognition.

To address the above issues, inspired by multi-scale feature extraction [15] and attention mechanisms [16], we propose a student facial expression recognition model based on multi-scale and deep fine-grained feature attention enhancement. The model captures and fuses different scales of facial information through the constructed multi-scale dual-pooling feature aggregation module to obtain a comprehensive representation of key facial features. Additionally, a key region-oriented attention mechanism is designed to focus on the nuances of facial expressions to further enhance the multi-scale deep fine-grained feature representation. Finally, the fusion of multi-scale and deep fine-grained attention-enhanced features is used to obtain richer and more accurate facial key information and achieve accurate facial expression recognition.

The main contributions of this paper are as follows:

1. A new multi-scale dual-pooling feature aggregation module is constructed, which captures and fuses facial information at different scales, thereby obtaining a comprehensive representation of key facial features.
2. A key region-oriented attention mechanism is designed, which enhances the ability to capture fine-grained features by directing key features, aiming to focus more on the nuances of facial expressions and less on the common areas of the face as a way to reduce the common interferences and thus improve the accuracy of recognition.
3. A smart classroom facial expression dataset (SCFED) during learning is created, and a large number of experiments are conducted on this dataset and four other public datasets, FER2013, FERPlus, AffectNet, and RAF-DB. The experimental results prove that the SFER-MDFAE proposed in this paper has certain advantages over the state-of-the-art facial expression recognition methods.

The rest of the paper is organized as follows: Section 2 briefly describes the related work of this paper. Section 3 describes the proposed method in detail. Section 4 shows the datasets and the comparison and analysis of experimental performance. Section 5 summarizes the work and gives an outlook.

## 2. Related Work

### 2.1. Facial Expression Recognition Based on Attention Mechanisms

With the development of computer vision technology, many researchers have begun to conduct facial expression recognition research in the field of deep learning. Jeong et al. [17] applied 3D convolution to extract spatial–temporal features, selected 23 facial landmarks to represent the movement of facial muscles, and finally used the designed joint classifier to output facial expression recognition results. Li et al. [18] proposed a novel method for facial cropping and rotation to preprocess input images and simplified the convolutional neural network, thereby improving the accuracy and speed of facial feature extraction. Fard et al. [19] proposed an Adaptive Correlation (Ad-Corre) loss, which guides the network to generate embedded feature vectors with high intra-class correlation and low inter-class correlation, thereby effectively enhancing the discriminative power of feature representations. Vignesh et al. [20] proposed a unique convolutional neural network that suppresses redundant information by embedding U-Net between VGG layers, allowing the model to extract more critical features.

The above studies mainly extracted expression features through a large amount of training data, without considering the varying importance of different facial regions in facial expression recognition. To address this limitation, researchers have incorporated attention mechanisms into facial expression recognition. Minaee et al. [21] proposed an approach based on attentional convolutional networks to capture facial key region features. Tang et al. [22] found that different facial regions contribute differently to expression recognition, and therefore proposed an approach combining bidirectional gated recurrent units (BiGRUs) with an attention mechanism. The BiGRUs model long-range dependencies of the region feature maps obtained using the window cropping strategy, while the attention mechanism adaptively adjusts the weight of each region, achieving precise facial expression recognition. Yu et al. [23] introduced the Channel Collaborative Attention Module (CCAM) and Spatial Collaborative Attention Module (SCAM) to accurately capture key facial expression features. Zheng et al. [16] proposed a cross-attention mechanism where landmark features and image features serve as mutual queries, reducing the attention to the common facial regions and highlighting the facial regions of interest. Although these methods have demonstrated excellent performance in facial expression recognition, they still fall short in extracting local fine-grained features in complex scenarios, leading to difficulties in distinguishing different classes of expressions with subtle differences. Consequently, improving the model’s ability to capture fine-grained features in complex environments remains an important direction for future research.

### 2.2. Facial Expression Recognition Based on Multi-Scale

Multi-scale facial expression feature extraction plays an important role in facial expression recognition. Fan et al. [24] systematically extracted kernel-scale information, network-scale information, and knowledge-scale information to enhance the representation of facial features of interest. Wen et al. [25] introduced a multi-head attention mechanism consisting of spatial and channel attention to address the limitations of a single attention head in simultaneously focusing on different facial regions. Here, spatial attention is used to capture features at multiple scales, and combining spatial attention with channel attention highlights subtle differences between expressions and reduces common interference, which leads to a more accurate categorization of expressions. Zhao et al. [26] utilized a multi-scale module to fuse features from different receptive fields, effectively reducing the sensitivity of deep convolution to pose variations. Karnati et al. [27] analyzed texture and residual features at different scales during feature extraction to capture more comprehensive facial expression information, which enhances the accuracy and robustness of facial expression recognition. Mao et al. [15] directly extracted three-layer multi-scale landmark features and three-layer multi-scale image features from the facial landmark detectors and image backbone, and added a small visual transformer network to fuse these features. The methods for extracting and fusing multi-scale features mentioned above effectively address the issue of scale variation. Consequently, in this paper, we propose a multi-scale dual-pooling feature aggregation method to extract and fuse key facial information at different scales, thereby enhancing the model’s recognition performance and robustness in various environments.

## 3. SFER-MDFAE Model

**Figure 1:**
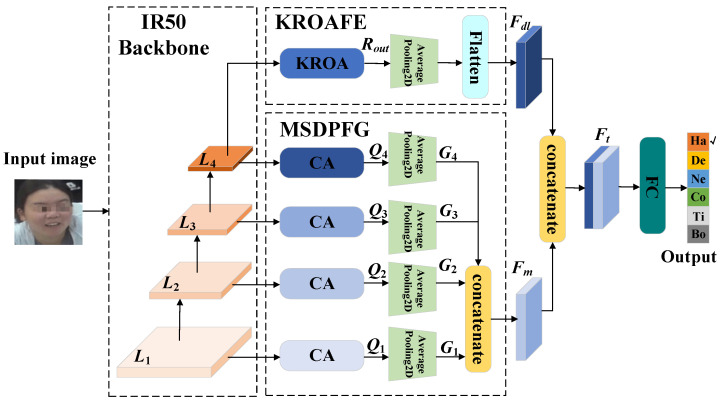
SFER-MDFAE model framework diagram.

The overall framework of the SFER-MDFAE model is shown in Figure 1. It mainly consists of four parts: the IR50 [28] backbone network, the multi-scale dual-pooling feature aggregation module (MDPFA), the key region-oriented attention feature enhancement module (KROAFE), and feature fusion. First, the given facial image is input into the IR50 backbone network to extract features at four different scales; second, these multi-scale features are input into the MDPFA module to obtain multi-scale channel attention aggregation features, which represent richer global semantic information and can improve the robustness of the model; then, the fourth layer of multi-scale features is input into the KROAFE module, where a key region-oriented attention mechanism captures high-dimensional deep fine-grained features, focusing on the nuances of facial expressions and reducing common interference; finally, the multi-scale channel attention aggregation features and the deep fine-grained features are fused to obtain richer and more accurate facial expression features, which are then input into the fully connected layer for facial expression classification.

### 3.1. Multi-Scale Feature Extraction

Existing facial expression recognition models mainly extract facial features at a single scale, neglecting factors such as lighting variations, viewpoint diversity, and distance differences between the target object and the camera, which can lead to the difficulty of the model in effectively capturing facial expression features at a single scale, thereby reducing the accuracy of facial expression recognition. To address the above issues, we employ IR50 as the backbone network to extract multi-scale features from the input images. The IR50 backbone network is composed of multiple different convolutional layers and pooling layers corresponding to different scales. Among them, convolution kernels of varying sizes have different receptive fields. Smaller convolution kernels mainly extract shallow image edge and texture information, while larger convolution kernels can capture a broader range of contextual information, thereby extracting deeper semantic features. Through this multi-scale feature extraction mechanism, IR50 can capture facial expression information at different layers. In addition, we propose two improvements based on related work [15,16,29]. First, the MobileNet backbone network used for extracting multi-scale landmark features is removed, retaining only the IR50 single backbone network. This avoids the problem of inaccurate landmark feature localization, further improves the accuracy, and simplifies the model structure. Second, multi-scale features $L_{1}$ , $L_{2}$ , $L_{3}$ , $L_{4}$ are extracted from the images using the IR50 backbone network. In particular, the fourth-layer high-dimensional feature $L_{4}$ is retained to better represent the deep semantic information of facial expressions and to further explore the subtle differences in facial expressions. Therefore, in the subsequent work, the multiscale features $L_{1}$ , $L_{2}$ , $L_{3}$ , $L_{4}$ are fed into the MDPFA module for extracting multiscale channel attention aggregation features, and the fourth-layer high-dimensional feature $L_{4}$ is fed into the KROAFE module to capture high-dimensional deep fine-grained attention-enhanced features.

### 3.2. Multi-Scale Dual-Pooling Feature Aggregation Module

To effectively capture comprehensive facial key features, we design a multi-scale dual-pooling feature aggregation module. The module mainly consists of two parts: channel attention feature selection and channel attention feature aggregation, which can effectively extract and fuse facial key information at different scales. This design significantly enhances the model’s recognition performance and robustness in different environments, thereby improving the accuracy and reliability of facial expression recognition.

#### 3.2.1. Channel Attention Feature Selection

**Figure 2:**
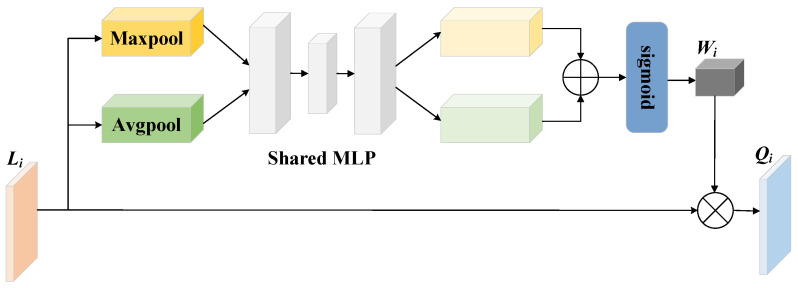
Structure of the channel attention mechanism. ⊕ represents addition, ⊗ represents multiplication.

The multi-scale features $L\in \left\{L_{1},L_{2},L_{3},L_{4}\right\}$ obtained by the IR50 backbone network still contain irrelevant or redundant information unrelated to facial expressions, and direct aggregation of these multiscale features will lead to an excessive number of features, which is not conducive to the processing of subsequent prediction layers. Therefore, a channel attention mechanism (CA) has been introduced. This mechanism dynamically adjusts the weights of channels to effectively capture shallow image edges, texture information, and deep semantic information across different scale features. By selectively extracting the most representative and effective expression information from each channel while minimizing information loss, it enhances the model’s feature representation capabilities and overall performance. The structure of the channel attention mechanism is shown in Figure 2.

Firstly, global maximum pooling and global average pooling are applied to the input facial expression features $L_{i}(i=1,2,3,4)$ at different scales to extract the most significant features and global average features in each channel; next, the features of each channel and their importance are learned through a shared multilayer perceptron (Shared MLP); then, the outputs of Shared MLP are summed up, and then the result after summing is mapped using the sigmoid activation function to obtain the channel attention weights $W_{i}(i=1,2,3,4)$ , as shown in Equation (1).

(1) $$W_{i}=sigmoid\left(MLP\left(MaxPool\left(L_{i}\right)\right)+MLP\left(AvgPool\left(L_{i}\right)\right)\right)$$

Here, MaxPool(⋅) refers to maximum average pooling and AvgPool(⋅) refers to global average pooling.

Finally, the input feature $L_{i}$ is multiplied by the channel attention weight $W_{i}$ to obtain the channel attention selection feature $Q_{i}(i=1,2,3,4)$ , as shown in Equation (2).

(2) $$Q_{i}=L_{i}\times W_{i}$$

#### 3.2.2. Channel Attention Feature Aggregation

The multi-scale features $L\in \left\{L_{1},L_{2},L_{3},L_{4}\right\}$ extracted from the IR50 backbone network are processed through the channel attention mechanism to obtain the channel attention selection features $Q\in \left\{Q_{1},Q_{2},Q_{3},Q_{4}\right\}$ . Subsequently, these features are individually fed into the global average pooling layer, which compresses the spatial dimensions of each channel into a single value; that is, the global average of each channel is computed to obtain $G\in \left\{G_{1},G_{2},G_{3},G_{4}\right\}$ . This is carried out to capture the global information of the entire image and reduces the spatial dimensions of the feature maps, thereby reducing the computational and parametric quantities of the model. To better leverage the key shallow image edges, texture features, and the deep semantic features from these different scale representations, a vector concatenation method is employed for their integration. This process generates a multi-scale channel attention aggregation feature $F_{m}$ that simultaneously captures both global visual information and fine-grained visual details, as illustrated in Equation (3).

(3) $$F_{m}=Concat\left(G_{1},G_{2},G_{3},G_{4}\right)$$

Here, Concat(⋅) refers to vector concatenation.

### 3.3. Key Region-Oriented Attention Feature Enhancement Module

**Figure 3:**
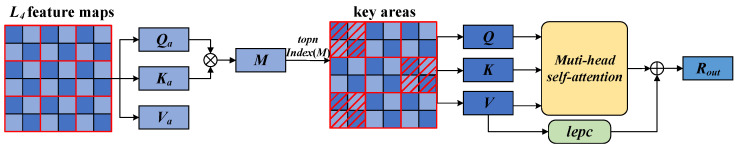
Structure of the key region-oriented attention mechanism. The blue squares represent patches, the red lines denote the divided regions, and the red italicized sections indicate the key regions.

In order to focus on the nuances of facial expressions in a more detailed way, reduce the focus on the common areas of the face, and reduce the impact of common interference on facial expression recognition, we propose a key region-oriented attention feature enhancement module. Unlike the related work [15], this paper eliminates the MobileNet backbone network. The reason for this removal is that the facial landmark features extracted by the MobileNet backbone are sometimes inaccurate, particularly in smart classroom settings where uneven lighting and diverse viewpoints can significantly affect the ability of the attention mechanism to capture crucial facial region information. Therefore, instead of using the landmark features extracted by MobileNet as prior knowledge for attention feature fusion with multi-scale facial features, the designed key region-oriented attention feature enhancement module is innovatively applied to the deep fine-grained feature $L_{4}$ . The deep feature map $L_{4}$ contains richer deep semantic information while reducing interference from shallow noise. This allows the key region-oriented attention mechanism (KROA) to focus more efficiently on fine-grained feature areas, thereby enhancing its ability to capture these fine-grained features. The design not only improves the accuracy of the model but also significantly reduces the computation and memory consumption of the model. The specific structure of KROA is shown in Figure 3.

Traditional attention mechanisms need to compute pairwise token interactions across all spatial locations, which generates a huge computational burden and memory usage; sparse attention mechanisms restrict attention operations to local windows [30], extended windows [31], and axially striped regions [32], which reduces computation and memory usage, but the key regions are not selected in a sufficiently accurate and flexible manner. In contrast to these methods, the key region-oriented attention mechanism dynamically selects the topn regions that are the most closely associated with each area and applies a multi-head self-attention mechanism among these regions. This approach enables more efficient extraction of critical facial expression features. The specific implementation method is as follows:

**Linear mapping:** For each patch in the input feature map $L_{4}\in {\mathbb{R}}^{H\times W\times C}$ ( C , H , and W refer to the number of channels, height, and width of the input feature map, respectively), linear transformations are applied to the queries, keys, and values, as shown in Equations (4)–(6).

(4) $$Q_{il}=L_{4}W_{il}^{q}$$

(5) $$K_{il}=L_{4}W_{il}^{k}$$

(6) $$V_{il}=L_{4}W_{il}^{v}$$

Here, i refers to the *i*-th patch; l refers to the *l*-th attention head; $Q_{il},K_{il},V_{il}\in {\mathbb{R}}^{H\times W\times C/l}$ refer to the query, key, and value matrices of each patch; and $W_{il}^{q},W_{il}^{k},W_{il}^{v}\in {\mathbb{R}}^{C\times C/l}$ refer to the weight matrices of $Q_{il},K_{il},V_{il}\in {\mathbb{R}}^{H\times W\times C/l}$ , respectively.

**Region division and mean calculation:** The feature map is divided into regions, and region-level queries and keys are computed based on all patches within each region. Specifically, the input feature map $L_{4}\in {\mathbb{R}}^{H\times W\times C}$ is divided into m×m non-overlapping regions so that each region contains H/m×W/m vectors and the mean values of the query and key of all patches in each region are used as the query and key of that region, as shown in Equations (7) and (8).

(7) $$Q_{al}^{j}=\frac{\sum_{i=1}^{HW/m^{2}}Q_{il}}{HW/m^{2}}$$

(8) $$K_{al}^{j}=\frac{\sum_{i=1}^{HW/m^{2}}K_{il}}{HW/m^{2}}$$

Here, *j* refers to the *j*-th region and $Q_{al}^{j},K_{al}^{j}\in {\mathbb{R}}^{m^{2}\times C/l}$ refers to the query and key of the *j*-th region. $HW/m^{2}$ represents the number of patches in each region.

**Correlation calculation and key region selection:** Different from the traditional sparse attention mechanism, this attention mechanism is more flexible and accurate for the selection of key regions. Specifically, a matrix multiplication operation is performed between the queries of each region and the key values of all other regions to calculate the degree of association $M\in {\mathbb{R}}^{m^{2}\times m^{2}}$ between the various regions. The topn regions with the highest degrees of association are retained for each region using the row indexing operator topIndex , as shown in Equations (9) and (10).

(9) $$M=Q_{\mathrm{al}}^{j}{\left(K_{\mathrm{al}}^{j}\right)}^{T}$$

(10) D=topnIndex(M)

In the equations, ${\left(\cdot \right)}^{T}$ refers to the matrix transposition and $D\in {\mathbb{N}}^{m^{2}\times n}$ represents the index of the topn most relevant regions for any query region. For example, the *j*-th row of $D\in {\mathbb{N}}^{m^{2}\times n}$ contains the indices of the topn regions that are the most relevant to the *j*-th region, where $1\leq topn\leq m^{2}$ , m=7 .

**Patch-to-patch attention:** After obtaining the index matrix $D\in {\mathbb{N}}^{m^{2}\times n}$ , each query patch in any query region j only focuses on all the patches within the topn regions indexed by $D\in {\mathbb{N}}^{m^{2}\times n}$ . Therefore, multi-head self-attention is performed only between all patches in region j and the key–value pairs within these topn related regions. The detailed process is shown in Equations (11)–(14).

(11) $$Attention\left(Q_{il},{K_{il}}^{\prime },{V_{il}}^{\prime }\right)=softmax\left(\frac{Q_{il}{\left({K_{il}}^{\prime }\right)}^{T}}{\sqrt{d}}\right){V_{il}}^{\prime }$$

(12) $${\mathrm{head}}_{l}=\mathrm{Attention}(L_{4}W_{\mathrm{il}}^{q},L_{4}{W_{\mathrm{il}}^{k}}^{\prime },L_{4}{W_{\mathrm{il}}^{q}}^{\prime })$$

(13) $$MHSA(Q_{\mathrm{il}}{K_{\mathrm{il}}}^{\prime }{V_{\mathrm{il}}}^{\prime })=Concat(head_{0},head_{1},\ldots ,head_{l})W^{o}$$

(14) $$R_{out}=MHSA\left(Q_{il},{K_{il}}^{\prime },{V_{il}}^{\prime }\right)+lpec\left({V_{il}}^{\prime }\right)$$

Here, Attention(⋅) refers to the single-head attention function and ${K_{il}}^{\prime },{V_{il}}^{\prime }\in {\mathbb{R}}^{nHW/m^{2}\times C/l}$ represent all the keys and values collected from the topn regions, respectively. softmax(⋅) refers to the normalization function, $\sqrt{d}$ refers to a scaling factor to prevent the gradient from disappearing, $head_{l}$ refers to the output attention score of the *l*-th attention head, and ${W_{il}^{k}}^{\prime }$ and ${W_{il}^{q}}^{\prime }$ represent the weight matrices for ${K_{il}}^{\prime }$ and ${V_{il}}^{\prime }$ , respectively. MHSA(⋅) refers to the output multi-head self-attention value; Concat(⋅) signifies vector concatenation, which aggregates the output attention scores from all attention heads to obtain the final attention score; $W^{O}$ is the weight matrix; $R_{out}$ refers to the output of the $L_{4}$ after applying the key region-oriented attention mechanism, and lpec(⋅) [33] refers to the local position enhancement encoding, which is essentially a deep downsampling convolution with a convolution kernel of 5.

After obtaining the attention feature $R_{out}$ , it is fed into a global average pooling layer, which aims to compress the spatial dimensions of each channel into a single value, that is, to compute the global average of each channel and thus extract the global average feature vector. Subsequently, the pooled features are flattened into a one-dimensional feature vector $F_{dl}$ , which represents the deep fine-grained attention-enhanced feature.

### 3.4. Feature Fusion

The multi-scale channel attention aggregation feature $F_{m}$ and the deep fine-grained attention-enhanced feature $F_{dl}$ are fused through vector concatenation. This fusion strategy not only obtains a comprehensive representation of the facial key features but also pays more attention to the nuances of different classes of expressions, thus obtaining richer and more accurate facial key information $F_{t}$ , as shown in Equation (15).

(15) $$F_{t}=Concat\left(F_{m},F_{dl}\right)$$

Here, Concat(⋅) refers to vector concatenation.

**Algorithm 1 illustrates the basic steps of SFER-MDFAE.**
**Algorithm 1:** SFER-MDFAE Algorithm
**Input:** Each batch contains 64 facial images from a smart classroom, with each image having a size of 
224×224
. **Output:** Predict the probability distribution for each image’s facial expression category (happy, dedicated, neutral, confused, tired, bored).
    1: Initialize the model architecture and its parameters.    2: **While** SFER-MDFAE Not Convergence **do**:    3:   Use the pre-trained IR50 as the backbone network to extract multi-scale features, namely $L_{1}$ , $L_{2}$ , $L_{3}$ , and $L_{4}$ .    4:   **While** i<4 **do**:    5:     For each layer of the multi-scale feature $L_{i}$ , selectively extract the most representative and effective facial expression information using the channel attention mechanism, resulting in the channel attention selection feature $Q_{i}$ .    6:     Perform global average pooling on the $Q_{i}$ to reduce the spatial dimensions of the channel, obtaining $G_{i}$ .    7:   **end while**    8:   Fuse the $G_{1}$ , $G_{2}$ , $G_{3}$ , and $G_{4}$ using vector concatenation to obtain the multi-scale channel attention aggregation feature $F_{m}$ .    9:   Linearly map the high-dimensional feature $L_{4}$ to queries, keys, and values, and perform down-sampling on values to obtain the local position enhancement feature.  10:   Calculate the similarity M between each query and key, and select the topn key regions index D based on the similarity.  11:   Collect the topn key–value pairs corresponding to the regions indicated by D .  12:   Use the multi-head attention mechanism to extract the key features and overlay the local positional enhancement features to finally obtain the attention feature $R_{out}$ .  13:   Perform global average pooling and flattening operations on $R_{out}$ to obtain the deep fine-grained feature $F_{dl}$ .  14:   Fuse the extracted $F_{m}$ and $F_{dl}$ features to obtain richer facial key information $F_{t}$ .  15:   Map the $F_{t}$ ; to the output space through a fully connected (FC) layer to obtain the probabilities for each class.  16: Compute the loss using the cross-entropy loss function and optimize the model using the gradient descent algorithm.  17: **end while**


## 4. Experiments

### 4.1. Datasets

To validate the advancement and effectiveness of the proposed SFER-MDFAE model, experiments are conducted on four classical facial expression datasets and one real smart classroom expression dataset, namely, the FER2013 [34], FERPlus [35], RAF-DB [36], AffectNet [37], and SCFEDs, respectively.

FER2013: The FER2013 dataset contains seven basic expressions (angry, disgust, fear, happy, sad, surprise, and neutral), with a total of 35,887 facial expression images, of which 28,709 are used for training, 3589 for validation, and 3589 for testing.

FERPlus: FERPlus is extended from FER2013 and reclassifies FER2013 into eight basic expressions (anger, contempt, disgust, fear, happy, neutral, sad, and surprise), which is more accurate and reasonable than FER2013. There are 31,341 facial expression images, of which 28,085 are used for training and 3256 for validation.

RAF-DB: The RAF-DB dataset contains seven basic expressions (surprise, fear, disgust, happy, sad, anger, and neutral), with a total of 15,339 facial expression images, of which 12,271 are used for training and 3068 for validation.

AffectNet: AffectNet is currently the largest publicly available facial expression dataset. AffectNet-7 includes seven basic expressions (neutral, happy, sad, surprise, fear, disgust, and anger), comprising a total of 287,401 facial images. Of these, 283,901 images are used for training, and 3500 are reserved for validation.

SCFED: The SCFED is captured using a SONY AX60 HD camera in a smart classroom scenario, containing six basic expressions (happy, dedicated, neutral, confused, tired, and bored), with a total of 36,226 facial expression images, of which 28,979 are used for training, 3619 for validation, and 3628 for testing. Specifically, for the high-resolution sequential images of students collected in a smart classroom environment, we employed the MTCNN (Multi-task Convolutional Neural Network) [38] algorithm for face detection and cropping, thereby obtaining individual facial images of the students. The expression labels were divided into six categories: happy, dedicated, neutral, confused, tired, and bored. During the annotation process, we invited several experts in education, psychology, and experienced teachers to manually label the facial images. If a particular expression label was agreed upon by more than 50% of the annotators, the image was classified under that label. Ultimately, we successfully constructed the SCFED.

### 4.2. Baseline Models and Evaluation Metrics

In order to evaluate the performance of the proposed SFER-MDFAE model, accuracy (ACC) is used as the evaluation metric, while the complexity of the model is assessed based on the number of parameters and computational cost, and the SFER-MDFAE model is compared with five state-of-the-art baseline models on five datasets, as detailed in Table 1.

### 4.3. Experimental Environment and Experimental Setup

The experimental environment of this paper is shown in Table 2.

The SFER-MDFAE model proposed in this paper uses different parameter settings for five different datasets.

FER2013: The learning rate is set to 1 × 10^−5^, the regularization coefficient is set to 1 × 10^−4^, the momentum is set to 0.9, and the batch size is set to 64.

FERPlus: The learning rate is set to 1 × 10^−5^, the regularization coefficient is set to 1 × 10^−4^, the momentum is set to 0.9, and the batch size is set to 100.

RAF-DB: The learning rate is set to 3 × 10^−5^, the regularization coefficient is set to 1 × 10^−4^, the momentum is set to 0.9, and the batch size is set to 64.

AffectNet: The learning rate is set to 2 × 10^−6^, the regularization coefficient is set to 5 × 10^−5^, the momentum is set to 0.9, and the batch size is set to 64.

SCFED: The learning rate is set to 1 × 10^−5^, the regularization coefficient is set to 1 × 10^−4^, the momentum is set to 0.9, and the batch size is set to 64.

Similar to POSTER [16], we also utilize the IR50 network pre-trained on the Ms-Celeb-1M [42] dataset as the backbone network. Gradient descent optimization is performed on all five datasets using the Adam optimizer with random level flipping and random erasure during training [43]. The number of topn regions retaining the maximum degree of the association is set to 15. In addition, to prevent overfitting, an early stopping strategy is used. For the FER2013, FERPlus, RAF-DB, and SCFEDs, training is stopped if the accuracy on the validation set does not improve for 50 consecutive epochs. For the AffectNet dataset, training is stopped if there is no improvement in validation accuracy for 12 consecutive epochs.

### 4.4. Experiments and Analysis of Results

**Figure 4:**
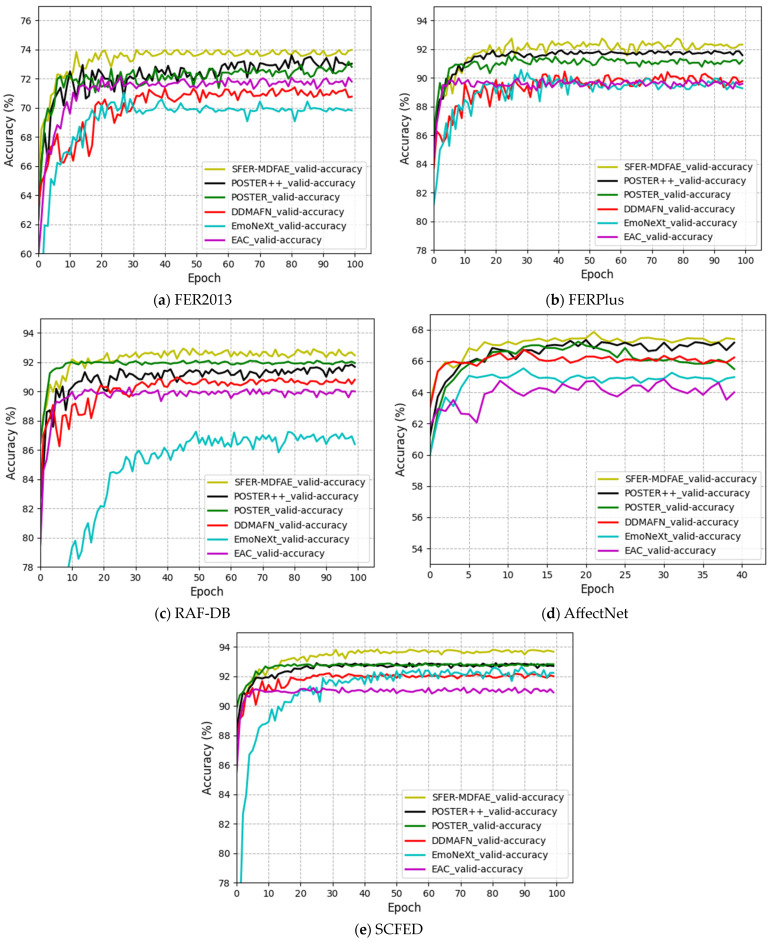
The accuracy curves of the validation sets for each model on five different datasets.

The accuracy curves of the validation sets for each model on the FER2013, FERPlus, RAF-DB, AffectNet, and SCFEDs are shown in Figure 4a–e.

As can be seen from Figure 4a–e, for both the FER2013 dataset and SCFED, the performance of the SFER-MDFAE model proposed in this paper outperforms the other baseline models after the 15th epoch of training and reaches convergence at the 30th epoch. For the FERPlus dataset, the SFER-MDFAE model outperforms the other baseline models after the 25th epoch of training and reaches convergence at the 30th epoch. For the RAF-DB dataset, the SFER-MDFAE model outperforms the other baseline models and reaches convergence after the 25th epoch of training. For the AffectNet dataset, the SFER-MFAE model outperforms the other baseline models after the fifth epoch of training and reaches convergence by the tenth epoch. It can be seen that the SFER-MDFAE model proposed in this paper shows fast convergence speed and excellent performance on the FER2013, FERPlus, RAF-DB, AffectNet, and SCFEDs, which indicates that the design and training strategy of the model has significant advantages, and is able to learn effective facial expression features in a shorter period of time with good generalization performance.

The performance metrics of the SFER-MDFAE model on the FER2013, FERPlus, RAF-DB, AffectNet, and SCFEDs, compared with other baseline models, are presented in Table 3. The computational complexity and the number of parameters for each model are shown in Table 4. The detailed analysis is as follows:

1. As can be seen from Table 3, the SFER-MDFAE model proposed in this paper significantly outperforms other existing methods on the FER2013 dataset. Specifically, the SFER-MDFAE achieves an accuracy of 76.18% on the FER2013 dataset, which represents improvements of 3.40%, 2.40%, 4.15%, 1.31%, and 1.45% over the EAC (72.78%), EmoNeXt (73.78%), DDAMFN (72.03%), POSTER (74.87%), and POSTER++ (74.73%) methods, respectively. These results indicate that despite the presence of some mislabeled data in the FER2013 dataset, the SFER-MDFAE model is still capable of achieving superior experimental outcomes.
2. As shown in Table 3, the SFER-MDFAE model proposed in this paper significantly outperforms other existing methods on the FERPlus dataset. Specifically, the SFER-MDFAE achieves an accuracy of 92.75% on the FERPlus dataset, representing improvements of 2.77%, 2.52%, 2.30%, 1.20%, and 0.83% over the EAC (89.98%), EmoNeXt (90.23%), DDAMFN (90.45%), POSTER (91.55%), and POSTER++ (91.92%) methods, respectively. Overall, the SFER-MDFAE model proposed in this paper shows a significant improvement over the state-of-the-art POSTER++ model, further demonstrating the superiority of our approach.
3. As can be seen from Table 3, the SFER-MDFAE model achieves an accuracy of 92.93% on the RAF-DB dataset, significantly outperforming other state-of-the-art methods. Specifically, the SFER-MDFAE model improves upon the EAC (90.16%), EmoNeXt (87.25%), DDAMFN (90.97%), POSTER (92.14%), and POSTER++ (92.17%) methods by 2.77%, 5.68%, 1.96%, 0.79%, and 0.76%, respectively. Compared to the FER2013 and FERPlus datasets, the RAF-DB dataset has a different distribution and quantity of facial expressions. The improvement of our model on the RAF-DB dataset further demonstrates its superior performance on public datasets and indicates its broad applicability.
4. As shown in Table 3, the accuracy of the SFER-MDFAE model on the AffectNet dataset reached 67.86%, significantly outperforming other state-of-the-art methods. Compared to EAC (64.74%), EmoNeXt (65.54%), DDAMFN (66.72%), POSTER (67.23%), and POSTER++ (67.34%), it achieved improvements of 3.12%, 2.32%, 1.14%, 0.63%, and 0.52%, respectively. These results demonstrate that the SFER-MDFAE model can achieve substantial performance gains even on large and complex datasets that are challenging to train on, further validating the model’s superiority and applicability.
5. As shown in Table 3, the SFER-MDFAE model performs exceptionally well on the SCFED, which represents the real smart classroom scenario. The model achieves an accuracy of 93.74%, outperforming other state-of-the-art methods. Compared to EAC (92.03%), EmoNeXt (92.42%), DDAMFN (93.00%), POSTER (93.25%), and POSTER++ (93.11%), the SFER-MDFAE model offers improvements of 1.71%, 1.32%, 0.74%, 0.49%, and 0.63%, respectively. Compared to other public datasets, in the smart classroom scenario, students’ expressions do not change significantly and are highly susceptible to occlusion by the environment and other factors, making it difficult for the model to recognize expressions. Despite these challenges, the SFER-MDFAE model still achieves a slight improvement over the advanced POSTER model. This indicates that the SFER-MDFAE model has a significant advantage in capturing facial expression features, particularly fine-grained features. Furthermore, its performance on the SCFED further validates the applicability and robustness of the SFER-MDFAE model in the specific context of smart classrooms.
6. As can be seen from Table 3 and Table 4, compared with the state-of-the-art POSTER and POSTER++ models, the SFER-MDFAE model significantly reduces the complexity of the model and the consumption of computational resources while maintaining a high level of accuracy and possesses the advantages of high efficiency and practicality.

**Figure 5:**
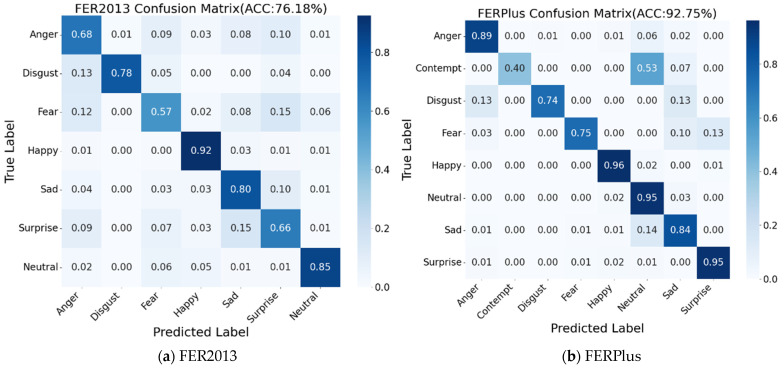

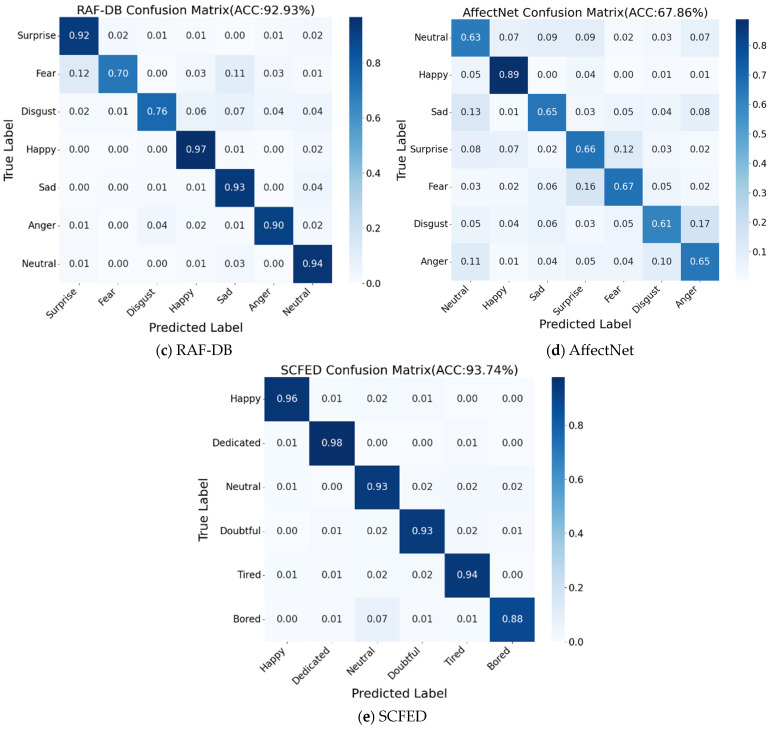
Confusion matrices of SFER-MDFAE model on five different datasets.

In this paper, the confusion matrix is used to evaluate the classification performance of the SFER-MDFAE model on various facial expression categories in order to exhaustively reveal the model’s recognition accuracy on different facial expression categories. By analyzing the confusion matrix, it is possible not only to quantify the overall recognition effectiveness of the model but also to gain insight into its correct classification and misclassification on each specific expression category. The confusion matrices of the SFER-MDFAE model on the FER2013, FERPlus, RAF-DB, AffectNet, and SCFEDs are shown in Figure 5a–e.

As indicated by the confusion matrix in Figure 5, the SFER-MDFAE model achieved the best performance in the “happy” expression category across the five expression datasets. This suggests that the model can relatively easily and accurately capture the facial features associated with “happy” expressions. In addition, the model shows high accuracy and low misclassification rate in recognizing all classes of expressions in the five expression datasets, which proves that the SFER-MDFAE model is able to capture the subtle changes in facial expressions and effectively differentiate between different classes of more similar expressions. Especially for the SCFED, the model does not recognize “dedicated” as “neutral” or “dedicated” as “neutral” when recognizing the two similar expressions, and the model shows very high accuracy in recognizing each class of expression. This shows that the SFER-MDFAE model is still able to efficiently recognize students’ learning expressions even in a complex classroom environment.

### 4.5. Results and Analysis of Ablation Experiments

**Figure 6:**
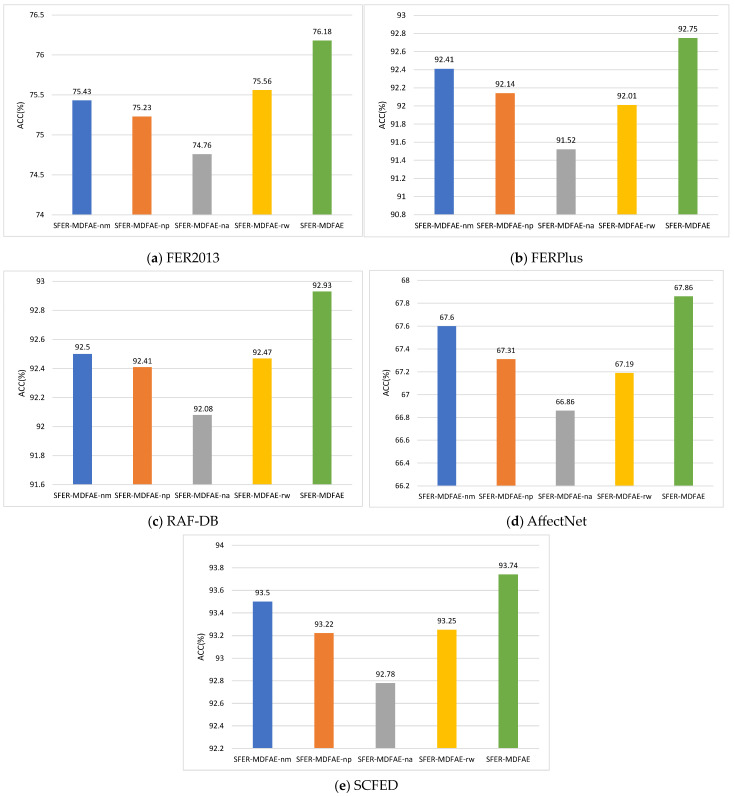
Results of ablation experiments of SFER-MDFAE model on five different datasets.

To validate the effectiveness of the multi-scale feature extraction, the multi-scale dual-pooling feature aggregation module, and the key region-oriented attention feature enhancement module in the SFER-MDFAE model, four variant models are designed and evaluated through ablation experiments across five datasets. Specifically, SFER-MDFAE-nm denotes the removal of multi-scale features $L_{1}$ , $L_{2}$ , $L_{3}$ , using only the deep semantic feature $L_{4}$ ; SFER-MDFAE-np indicates the removal of the multi-scale dual-pooling feature aggregation module; SFER-MDFAE-na represents the removal of the key region-oriented attention mechanism; and SFER-MDFAE-rw replaces the proposed key region-oriented attention mechanism with a window-based cross-attention mechanism [13]. The experimental results are shown in Figure 6a–d.

As can be seen from the ablation experiment results in Figure 6, the accuracy of SFER-MDFAE-nm is significantly reduced compared to that of SFER-MDFAE, indicating the importance of multi-scale features for a comprehensive understanding of faces. By capturing facial expression features at different scales in the image, the model can more effectively comprehend shallow edge and texture features and deep semantic features present in expression images. This multi-scale approach significantly improves the model’s feature expression ability, leading to improved recognition accuracy; the accuracy of SFER-MDFAE-np is lower than that of SFER-MDFAE, indicating the effectiveness of the proposed multi-scale dual-pooling feature aggregation module. This module extracts effective features at different scales through the channel feature selection mechanism and fuses them to obtain comprehensive representations of key facial features, which helps the model maintain high recognition accuracy even when recognizing noisy expression images; the accuracy of SFER-MDFAE surpasses that of its two variant models, SFER-MDFAE-na and SFER-MDFAE-rw, indicating that the proposed key region-oriented attention mechanism is not only effective but also superior to the traditional window-based cross-attention mechanism. This advantage arises because the key region-oriented attention mechanism dynamically selects the most relevant regions for each key area and applies a multi-head self-attention mechanism between these regions. This allows the model to accurately capture the global correlations between regions, rather than being limited to information within a local window, thus improving the extraction of key facial expression features. Furthermore, removing any of the modules leads to a decrease in model performance, which suggests that on all five datasets, all three modules introduced are valid and play a complementary role in the model.

### 4.6. Parametric Analysis

**Figure 7:**
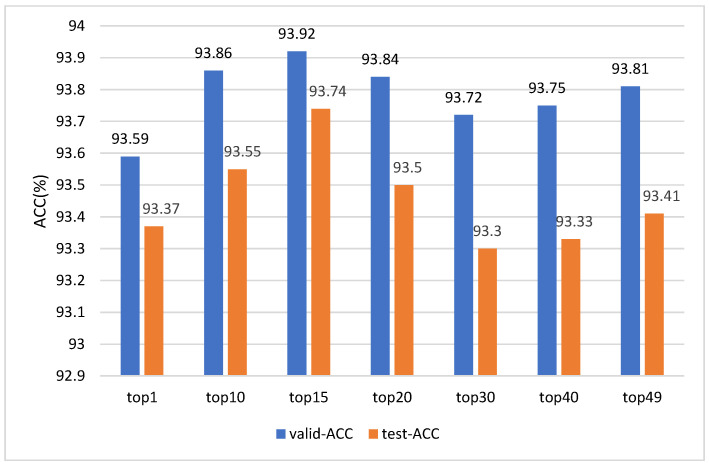
Validation set accuracy and test set accuracy of the SFER-MDFAE model at different topn values.

In order to verify the influence of the value of the number of topn , which retains the number of regions with the maximum degree of association, on the SFER-MDFAE model, we conducted experiments with different values of topn ( topn=1,10,15,20,30,40,49 ). Since the experimental results are consistent across datasets, we analyze the smart classroom facial expression dataset SCFED as an example, and the results are shown in Figure 7.

As can be seen from Figure 7, the value of topn has a significant effect on the validation set accuracy and test set accuracy of the model. The model performs best when topn=15 , reaching 93.92% accuracy in the validation set and 93.74% in the test set. Lower topn values (e.g., topn=1 ) do not allow the model to adequately capture the deep semantic information of facial features, resulting in insufficient attention to the nuances of facial expressions, which in turn affects the performance of the model. Higher topn values (e.g., topn=30,40,49 ), on the other hand, may reintroduce noise, causing the model to pay too much attention to common regions of different classes of facial expressions, which in turn reduces the effectiveness of the key region-oriented attention mechanism. Therefore, an appropriate topn value allows the model to focus more on the nuances of facial expressions and less on the common regions of the face, thereby improving the model’s performance.

### 4.7. Visualization Analysis

**Figure 8:**
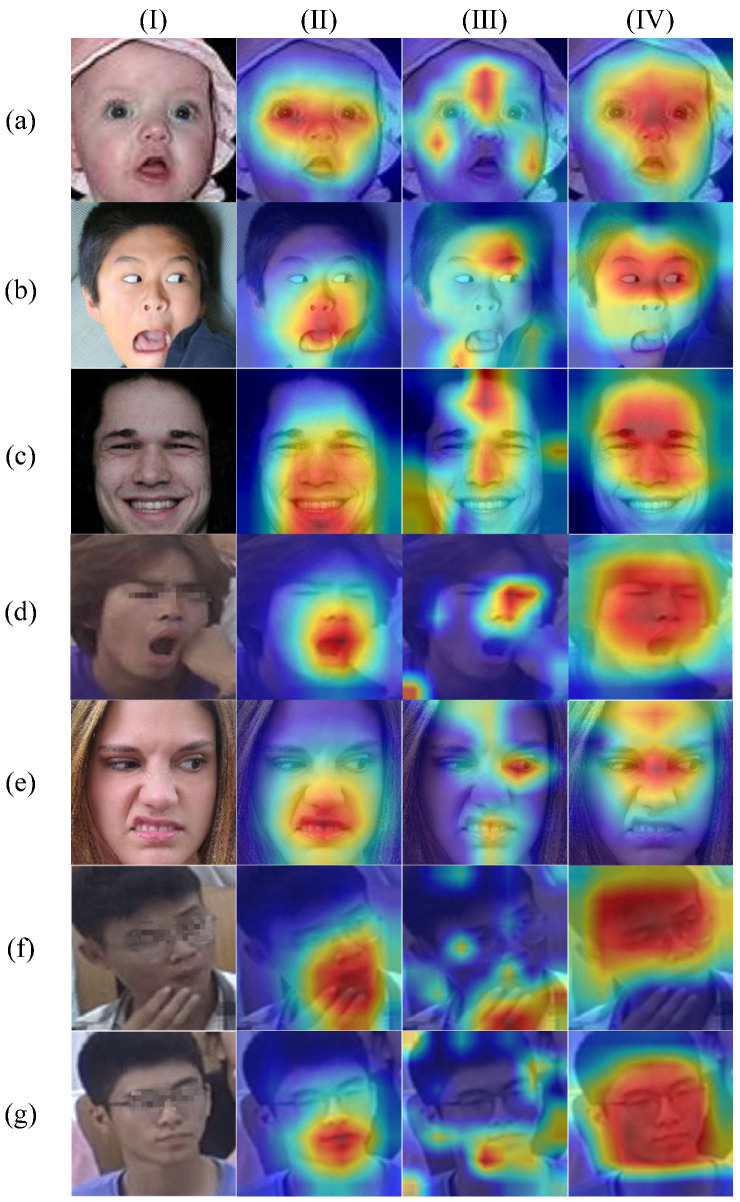
Examples of attention visualization of various facial expression images from the RAF-DB and SCFEDs. Rows (**a**–**g**) represent surprise, fear, happy, tired, disgust, doubt, and neutral, respectively. (**I**) Original image; (**II**) attention visualization results for the fourth layer of high-dimensional feature $L_{4}$ ; (**III**) attention visualization results output by the baseline model with window-based cross-attention mechanism; (**IV**) attention visualization results output by the KROAFE module.

In order to better explain the effect of the key region-oriented attention mechanism in the SFER-MDFAE model, we randomly selected seven different kinds of facial images from the RAF-DB and SCFEDs and used the GradCAM [44] visualization method to generate the attention heatmaps, and the results are shown in Figure 8. In Figure 8, rows (a)–(g) represent seven classes of expressions: surprise, fear, happy, tired, disgust, doubt, and neutral, respectively. Column (I) is the original facial image, column (II) is the attention visualization result of the fourth layer of high-dimensional features $L_{4}$ output by IR50, column (III) is the attention visualization result output by the baseline model [15] based on the window-based cross-attention mechanism, and column (IV) is the attention visualization result output by the KROAFE module proposed in this paper.

As can be seen from Figure 8, although column (II) effectively recognizes the facial region in the original images, it is unable to recognize the fine-grained features of the face, which has some limitations. For example, in the category of disgusted expressions in row (e) of Figure 8, column (II) only recognizes the universal regions and fails to effectively capture the key regions that help recognize disgusted expressions, such as the forehead and between the eyebrows. In contrast, column (IV) shows that KROAFE is able to accurately focus on the key regions ignored in column (II), including eyebrows, forehead, eyes, and mouth, etc., and the attention result is significantly better than that in column (II), which suggests that KROAFE is able to efficiently extract the fine-grained features of a complex face. It is worth noting that the attention regions in column (IV) are larger due to the fact that KROAFE residually joins the extracted fine-grained feature with the downsampled feature $L_{4}$ . From another perspective, the attention features extracted in column (III) are less stable compared to those in the fourth column (IV), and column (III) often focuses on non-facial invalid regions in complex facial expressions, as shown in rows (c), (f), and (g). In particular, in the confused expression category in row (f), due to the presence of the hand interference at the bottom, column (III) incorrectly focuses on the hand interference region instead of the face region, which may lead to a final misjudgment. Although this is an isolated case, such instances may have a significant impact on the overall performance of the model in the complex environments of a real-world smart classroom. On the contrary, column (IV) is not affected by the interfering regions, and the overall visualization of the face in the examples is significantly better than column (III). This is not only due to the superiority of the KROAFE module but also due to the fact that the MobileNet network is removed in this paper, which avoids the problem of inaccurate landmark feature localization leading to inaccurate extraction of key regions. In summary, the above results further validate the effectiveness of the KROAFE method proposed in this paper.

## 5. Conclusions

In this paper, we propose a student facial expression recognition model based on multi-scale and deep fine-grained feature attention enhancement. The facial expression information at different scales is captured and fused through a multi-scale dual-pooling feature aggregation module as a way to obtain a comprehensive representation of key facial features and improve the robustness of the model. In addition, a key region-oriented attention mechanism is designed to pay more attention to the nuances of facial expressions, further enhancing the representation of multi-scale deep fine-grained features. Compared with the traditional window-based cross-attention mechanism, this mechanism can dynamically select and focus on key regions in the image without the limitation of a fixed window, enhancing the expression of facial features. The multi-scale and deep fine-grained attention-enhanced features are fused to obtain richer and more accurate facial key information and realize the accurate classification of facial expressions. The experimental results on five datasets demonstrate that the SFER-MDFAE model proposed outperforms the other five baseline models. In addition, this paper conducts a large number of ablation experiments to demonstrate the effectiveness of the multi-scale feature extraction, multi-scale dual-pooling feature aggregation module, and the key region-oriented attention feature enhancement module. In future research, we will focus on further optimizing the key region-oriented attention mechanism to enhance its accuracy and efficiency in capturing complex emotional features. Meanwhile, we aim to promote facial expression recognition technology in smart classrooms, integrating information such as students’ head poses to better characterize their learning emotions. This will assist teachers in evaluating students’ learning states more effectively. Additionally, as our research group’s plans deepen and expand, we intend to utilize virtual reality (VR) technology to simulate diverse smart teaching scenarios and incorporate it into wearable devices. This approach aims to better address the varied demands of smart education and facilitate the widespread application of emotion recognition technology.

## Figures and Tables

**Table 1:** Baseline models.

Baseline Models	Model Introduction
EAC [39]	An erasure attention consistency method is proposed to effectively suppress noisy samples during training by randomly erasing images and exploiting flip semantic consistency.
* EmoNeXt [40]	An adaptive facial expression recognition model is proposed, which optimizes feature extraction by integrating a spatial transformer network and squeeze-and-excitation blocks. Additionally, a self-attention regularization term is introduced to generate compact feature vectors.
DDAMFN [41]	Hybrid feature networks and bidirectional attention networks are proposed to effectively extract features and capture remote dependencies.
POSTER [16]	A transformer-based dual-stream pyramid cross-fusion method is proposed, which effectively integrates facial landmark features and image features, thereby maximizing attention to prominent facial areas.
POSTER++ [15]	A window-based cross-attention mechanism is proposed, and the branch from the image to landmarks is removed in the dual-stream design, achieving effective recognition with lower computational cost.

* The baseline model referred to as EmoNext is the EmoNext-base model.

**Table 2:** Experimental environment.

Experimental Environment	Environment Configuration
Operating systems	Linux
CPU	Intel(R) Core (TM) i5-13490F
Video Cards	GeForce RTX 4060Ti
RAM	16 GB
ROM	2T SSD
Programming Languages	Python 3.8
Framework	Pytorch

**Table 3:** Comparison of the SFER-MDFAE with baseline models on each dataset.

Dataset	Model	Accuracy (%)
FER2013	EAC	72.78
	EmoNeXt	73.78
	DDAMFN	72.03
	POSTER	74.87
	POSTER++	74.73
	**SFER-MDFAE**	76.18
FERPlus	EAC	89.98
	EmoNeXt	90.23
	DDAMFN	90.45
	POSTER	91.55
	POSTER++	91.92
	**SFER-MDFAE**	92.75
RAF-DB	EAC	90.16
	EmoNeXt	87.25
	DDAMFN	90.97
	POSTER	92.14
	POSTER++	92.17
	**SFER-MDFAE**	92.93
AffectNet	EAC	64.74
	EmoNeXt	65.54
	DDAMFN	66.72
	POSTER	67.23
	POSTER++	67.34
	**SFER-MDFAE**	67.86
SCFED	EAC	92.03
	EmoNeXt	92.42
	DDAMFN	93.00
	POSTER	93.25
	POSTER++	93.11
	**SFER-MDFAE**	93.74

**Table 4:** Comparison of FLOPs and Params between the SFER-MDFAE model and baseline models.

**Model**	**FLOPs**	**Params**
EAC	3.898 G	23.522 M
EmoNeXt	15.438 G	91.708 M
DDAMFN	0.564 G	4.106 M
POSTER	7.895 G	58.976 M
POSTER++	8.482 G	43.724 M
**SFER-MDFAE**	**6.362 G**	**31.975 M**

## Data Availability

The datasets are available at the following link: FER2013: https://www.kaggle.com/c/challenges-in-representation-learning-facial-expression-recognition-challenge/data (accessed on 1 October 2024), FERPlus: https://www.worldlink.com.cn/osdir/ferplus.html (accessed on 1 October 2024), RAF-DB: http://www.whdeng.cn/RAF/model1.html (accessed on 1 October 2024), AffectNet: http://mohammadmahoor.com/affectnet/ (accessed on 1 October 2024). The SCFED will be made available upon reasonable request.

## References

[1] Goldberg P., Sümer Ö., Stürmer K., Wagner W., Göllner R., Gerjets P., Kasneci E., Trautwein U. (2021). Attentive or not? Toward a machine learning approach to assessing students’ visible engagement in classroom instruction. Educ. Psychol. Rev..

[2] Munna A.S., Kalam M.A. (2021). Teaching and learning process to enhance teaching effectiveness: A literature review. Int. J. Humanit. Innov. (IJHI).

[3] Castiblanco Jimenez I.A., Olivetti E.C., Vezzetti E., Moos S., Celeghin A., Marcolin F. (2024). Effective affective EEG-based indicators in emotion-evoking VR environments: An evidence from machine learning. Neural Comput. Appl..

[4] Marín-Morales J., Higuera-Trujillo J.L., Greco A., Guixeres J., Llinares C., Scilingo E.P., Alcañiz M., Valenza G. (2018). Affective computing in virtual reality: Emotion recognition from brain and heartbeat dynamics using wearable sensors. Sci. Rep..

[5] Saurav S., Saini R., Singh S. (2023). Fast facial expression recognition using boosted histogram of oriented gradient (BHOG) features. Pattern Anal. Appl..

[6] Shi Y., Lv Z., Bi N., Zhang C. (2020). An improved SIFT algorithm for robust emotion recognition under various face poses and illuminations. Neural Comput. Appl..

[7] Niu B., Gao Z., Guo B. (2021). Facial expression recognition with LBP and ORB features. Comput. Intell. Neurosci..

[8] Lakshmi D., Ponnusamy R. (2021). Facial emotion recognition using modified HOG and LBP features with deep stacked autoencoders. Microprocess. Microsyst..

[9] Shou Z., Zhu N., Wen H., Liu J., Mo J., Zhang H. (2023). A Method for Analyzing Learning Sentiment Based on Classroom Time-Series Images. Math. Probl. Eng..

[10] Xue F., Tan Z., Zhu Y., Ma Z., Guo G. Coarse-to-fine cascaded networks with smooth predicting for video facial expression recognition. Proceedings of the IEEE/CVF Conference on Computer Vision and Pattern Recognition.

[11] Nan Y., Ju J., Hua Q., Zhang H., Wang B. (2022). A-MobileNet: An approach of facial expression recognition. Alex. Eng. J..

[12] Farzaneh A.H., Qi X. Facial expression recognition in the wild via deep attentive center loss. Proceedings of the IEEE/CVF Winter Conference on Applications of Computer Vision.

[13] Zhang Y., Li Y., Liu X., Deng W. (2024). Leave no stone unturned: Mine extra knowledge for imbalanced facial expression recognition. Adv. Neural Inf. Process. Syst..

[14] Yu J., Wei Z., Cai Z., Zhao G., Zhang Z., Wang Y., Xie G., Zhu J., Zhu W., Liu Q. Exploring Facial Expression Recognition through Semi-Supervised Pre-training and Temporal Modeling. Proceedings of the IEEE/CVF Conference on Computer Vision and Pattern Recognition.

[15] Mao J., Xu R., Yin X., Chang Y., Nie B., Huang A., Wang Y. (2023). POSTER++: A simpler and stronger facial expression recognition network. arXiv.

[16] Zheng C., Mendieta M., Chen C. Poster: A pyramid cross-fusion transformer network for facial expression recognition. Proceedings of the IEEE/CVF International Conference on Computer Vision.

[17] Jeong D., Kim B.G., Dong S.Y. (2020). Deep joint spatiotemporal network (DJSTN) for efficient facial expression recognition. Sensors.

[18] Li K., Jin Y., Akram M.W., Han R., Chen J. (2020). Facial expression recognition with convolutional neural networks via a new face cropping and rotation strategy. Vis. Comput..

[19] Fard A.P., Mahoor M.H. (2022). Ad-corre: Adaptive correlation-based loss for facial expression recognition in the wild. IEEE Access.

[20] Vignesh S., Savithadevi M., Sridevi M., Sridhar R. (2023). A novel facial emotion recognition model using segmentation VGG-19 architecture. Int. J. Inf. Technol..

[21] Minaee S., Minaei M., Abdolrashidi A. (2021). Deep-emotion: Facial expression recognition using attentional convolutional network. Sensors.

[22] Tang C., Zhang D., Tian Q. (2023). Convolutional Neural Network–Bidirectional Gated Recurrent Unit Facial Expression Recognition Method Fused with Attention Mechanism. Appl. Sci..

[23] Yu W., Xu H. (2022). Co-attentive multi-task convolutional neural network for facial expression recognition. Pattern Recognit..

[24] Fan X., Jiang M., Shahid A.R., Yan H. (2022). Hierarchical scale convolutional neural network for facial expression recognition. Cogn. Neurodyn..

[25] Wen Z., Lin W., Wang T., Xu G. (2021). Distract your attention: Multi-head cross attention network for facial expression recognition. arXiv.

[26] Zhao Z., Liu Q., Wang S. (2021). Learning deep global multi-scale and local attention features for facial expression recognition in the wild. IEEE Trans. Image Process..

[27] Karnati M., Seal A., Yazidi A., Krejcar O. (2022). Flepnet: Feature level ensemble parallel network for facial expression recognition. IEEE Trans. Affect. Comput..

[28] Deng J., Guo J., Xue N., Zafeiriou S. Arcface: Additive angular margin loss for deep face recognition. Proceedings of the IEEE/CVF Conference on Computer Vision and Pattern Recognition.

[29] Xu R., Huang A., Hu Y., Feng X. (2023). GFFT: Global-local feature fusion transformers for facial expression recognition in the wild. Image Vis. Comput..

[30] Liu Z., Lin Y., Cao Y., Hu H., Wei Y., Zhang Z., Lin S., Guo B. Swin transformer: Hierarchical vision transformer using shifted windows. Proceedings of the IEEE/CVF International Conference on Computer Vision.

[31] Tu Z., Talebi H., Zhang H., Yang F., Milanfar P., Bovik A., Li Y. (2022). Maxvit: Multi-axis vision transformer. Proceedings of the European Conference on Computer Vision.

[32] Wang W., Chen W., Qiu Q., Chen L., Wu B., Lin B., He X., Liu W. (2024). Crossformer++: A versatile vision transformer hinging on cross-scale attention. IEEE Trans. Pattern Anal. Mach. Intell..

[33] Ren S., Zhou D., He S., Feng J., Wang X. Shunted self-attention via multi-scale token aggregation. Proceedings of the IEEE/CVF Conference on Computer Vision and Pattern Recognition.

[34] Goodfellow I.J., Erhan D., Carrier P.L., Courville A., Mirza M., Hamner B., Cukierski W., Tang Y., Thaler D., Lee D.H. (2013). Challenges in representation learning: A report on three machine learning contests. Proceedings of the Neural Information Processing: 20th International Conference, ICONIP 2013.

[35] Barsoum E., Zhang C., Ferrer C.C., Zhang Z. Training deep networks for facial expression recognition with crowd-sourced label distribution. Proceedings of the 18th ACM International Conference on Multimodal Interaction.

[36] Li S., Deng W. (2020). Deep facial expression recognition: A survey. IEEE Trans. Affect. Comput..

[37] Mollahosseini A., Hasani B., Mahoor M.H. (2017). Affectnet: A database for facial expression, valence, and arousal computing in the wild. IEEE Trans. Affect. Comput..

[38] Zhang K., Zhang Z., Li Z., Qiao Y. (2016). Joint face detection and alignment using multitask cascaded convolutional networks. IEEE Signal Process. Lett..

[39] Zhang Y., Wang C., Ling X., Deng W. (2022). Learn from all: Erasing attention consistency for noisy label facial expression recognition. Proceedings of the European Conference on Computer Vision.

[40] El Boudouri Y., Bohi A. EmoNeXt: An Adapted ConvNeXt for Facial Emotion Recognition. Proceedings of the 2023 IEEE 25th International Workshop on Multimedia Signal Processing (MMSP).

[41] Zhang S., Zhang Y., Zhang Y., Wang Y., Song Z. (2023). A dual-direction attention mixed feature network for facial expression recognition. Electronics.

[42] Guo Y., Zhang L., Hu Y., He X., Gao J. (2016). Ms-celeb-1m: A dataset and benchmark for large-scale face recognition. Proceedings of the Computer Vision–ECCV 2016: 14th European Conference.

[43] Zhong Z., Zheng L., Kang G., Li S., Yang Y. Random erasing data augmentation. Proceedings of the AAAI Conference on Artificial Intelligence.

[44] Selvaraju R.R., Cogswell M., Das A., Vedantam R., Parikh D., Batra D. Grad-cam: Visual explanations from deep networks via gradient-based localization. Proceedings of the IEEE International Conference on Computer Vision.

